# Osteopontin Preconditioning Improves the Regenerative Effects of Mesenchymal Stem Cells In Vitro but Not Their Therapeutic Efficacy Following Hypoxia-Ischemia in Mice

**DOI:** 10.3390/cells14221824

**Published:** 2025-11-20

**Authors:** Sara T. De Palma, Celine N. van Wijk-Eeftink, Lisanne M. Baak, Cora H. A. Nijboer, Caroline G. M. de Theije

**Affiliations:** 1 Department for Developmental Origins of Disease, University Medical Center Utrecht Brain Center and Wilhelmina Children’s Hospital, Utrecht University, 3584 EA Utrecht, The Netherlands; s.depalma-2@umcutrecht.nl (S.T.D.P.); c.nijboer@umcutrecht.nl (C.H.A.N.); 2 Department of Neonatology, University Medical Center Utrecht Brain Center and Wilhelmina Children’s Hospital, Utrecht University, 3584 EA Utrecht, The Netherlands

**Keywords:** hypoxic-ischemic brain injury, neonate, mesenchymal stem cells, preconditioning, osteopontin, intranasal

## Abstract

Hypoxic-ischemic (HI) brain injury is associated with high mortality and severe long-term neurodevelopmental impairments in term newborns. Intranasal mesenchymal stem cell (MSC) therapy is a promising strategy to boost neurorepair after injury, and optimization strategies to further enhance its therapeutic potential are under development. In this study, we explored whether 24 h preconditioning of MSCs with 1000 ng/mL of osteopontin (OPN) could enhance MSC properties in vitro and in vivo. OPN-preconditioned MSCs (OPN-MSCs) showed increased activation of the ERK transcription pathway at 1 h during preconditioning and enhanced migration compared to naïve-MSCs. OPN preconditioning also altered gene expression of neurotrophic and immunomodulatory factors in MSCs. In vitro assessment of MSC potency showed that while OPN-MSCs were as effective as naïve-MSCs in reducing microglia activation, OPN preconditioning enhanced the potency of MSCs to boost neural stem cell differentiation into more complex neurons. However, in vivo, OPN-MSCs were not superior to naïve-MSCs in reducing lesion size in mice when applied at 3 days post-HI. Altogether, OPN preconditioning enhanced the migratory and neurotrophic properties of MSCs in vitro but not in vivo, highlighting its potential to optimize MSC function while underscoring the need for further research to refine in vivo translation and to evaluate functional outcomes for therapeutic efficacy.

## 1. Introduction

Hypoxic-ischemic encephalopathy (HIE), caused by perinatal asphyxia, is a severe clinical condition associated with high mortality and morbidity in term-born infants [1,2]. The life-long consequences of newborns suffering from HIE are dependent on the timing and duration of the insult and can include cerebral palsy, cognitive and behavioral disabilities, as well as verbal impairments and difficulties with social tasks [3,4,5,6]. At the moment, the only clinically validated therapy for neonates with moderate to severe HIE is therapeutic hypothermia [7,8]. However, therapeutic hypothermia has shown to be only modestly effective in protecting the brain from hypoxic-ischemic (HI) injury and can only be applied within a narrow treatment window of 6 h after the insult [8]. Therefore, novel therapeutic strategies are needed to overcome the limitations of currently available treatment options.

In the last decade, stem-cell-based therapies, particularly with mesenchymal stem cells (MSCs), have come forward as an alternative approach aimed at repairing the neonatal brain after injury. Intranasal administration of MSCs has shown to mitigate brain damage and improve sensorimotor and cognitive function in experimental rodent models of neonatal HI brain injury, even when applied as late as 10 days after injury [9,10,11,12]. Upon intranasal delivery, MSCs migrate to the lesion site in the brain [9,11], where they come into contact with a plethora of proteins, including growth factors, cytokines, and chemokines released by the HI-injured tissue in the hours and days after the insult [13]. MSCs respond to this microenvironment by, in turn, secreting more growth, immunomodulatory, and trophic factors that can aid in modulating inflammation and promoting endogenous regeneration of the neonatal brain [14]. Importantly, our group has recently demonstrated the safety and feasibility of intranasal MSC treatment in 10 term-born neonates with HI brain injury caused by perinatal stroke in the first-in-human phase I clinical trial, with promising first results on motor outcome at 2-year follow-up [15,16]. Despite these promising results, intranasal MSC therapy has been shown to reduce lesion size by ±30–50% in rodent models of HI injury [11], leaving a window to enhance its therapeutic efficacy. Preconditioning of MSCs has emerged as a promising strategy to boost their therapeutic potential [17], for example using hypoxic cultures [18]. However, preconditioning with a defined agent, such as a recombinant protein, may be simpler to standardize and scale under good manufacturing practice and could produce a more homogeneous, optimized MSC product. Preconditioning with cytokines (e.g., IFN-γ or IL-1) has been shown to modulate the immunomodulatory mechanisms of MSCs [19,20]. Preconditioning agents that target the migratory and neurotrophic mechanisms of MSCs are understudied.

Secreted phosphoprotein 1 (SPP1), also known as osteopontin (OPN), is a multifunctional glycoprotein, upregulated in organs in response to inflammation and injury [21,22,23]. In the brain, OPN is predominantly expressed by microglia and astrocytes [24,25], and in a previous study, our group showed that HI brain damage is exacerbated in neonatal mice deficient in OPN compared to wild-type littermates, thus implying a neuroprotective role for OPN [26]. Additionally, OPN has been shown to modulate injury and repair in several brain pathologies such as multiple sclerosis, Parkinson’s disease, brain tumors, and stroke [24,25,27,28,29]. Similarly, in vitro studies have implicated a positive effect of OPN on the neurogenic process through enhanced induction of neuronal proliferation, differentiation, and migration [30,31,32]. Moreover, intracerebroventricular or intranasal administration of OPN after stroke and subarachnoid hemorrhage in adult rodent models has shown to reduce infarct size and brain edema [33,34,35,36,37] and to reduce brain inflammation after intracerebral hemorrhage in hyperglycemic rats [38]. After binding to its receptors, i.e., integrins and isoforms of CD44, OPN activates various intracellular signaling pathways that result in cell adhesion, chemotaxis, and differentiation, as well as the secretion of factors involved in angiogenesis and immunomodulation [39,40,41,42]. Supplementation of OPN has been shown to improve the angiogenic properties of endothelial cells and enhance proliferation of human bone-marrow–derived MSCs [38]; similarly, OPN restored proliferative capacity of senescent adipose-derived MSCs in an in vitro model and promoted osteogenic differentiation [43]. Recent studies have also identified OPN as a potent inducer of MSC migration, mediated through the extracellular-signal-regulated kinase (ERK) pathway [44,45,46]. Given the importance of both migration and secretome-driven mechanisms in MSC-based therapies for neonatal HI brain injury, the aim of this study was to investigate whether preconditioning of MSCs to OPN prior to intranasal administration could improve their therapeutic efficacy. First, we assessed whether MSCs expressed OPN receptors and whether they could be activated by OPN exposure. Second, we studied the in vitro migration of OPN-preconditioned MSCs (OPN-MSCs). We further explored how OPN preconditioning affects the expression of immunomodulatory and neurotrophic factors in MSCs and assessed whether OPN-MSCs are more effective in dampening in vitro neuroinflammation and providing neurotrophic support. Lastly, we investigated the therapeutic efficacy of intranasally administered OPN-MSCs in a neonatal mouse model of HI brain injury and compared them to naïve-MSCs.

## 2. Materials and Methods

### 2.1. Ethical Approval

All procedures were carried out according to the Dutch and European international guidelines (Directive 86/609, ETS 123, Annex II) and were approved by the Central Authority for Scientific Procedures on Animals (project license: AVD11500202115334) and the local Experimental Animal Committee Utrecht. The experiments are reported in compliance with the ARRIVE guidelines [47].

### 2.2. Mesenchymal Stem Cell Culture and Osteopontin Preconditioning

GIBCO^®^ mouse (C57BL/6) bone-marrow-derived mesenchymal stem cells (MSCs, #S1502-100, Invitrogen, Thermo Fisher Scientific, Waltham, MA, USA) of passage 8 were cultured in T75 flasks (#353110, Corning, Somerville, MA, USA) in Dulbecco’s Modified Eagle Medium with GlutaMAX (DMEM:F12, #31331093, Gibco^TM^, Thermo Fisher Scientific) supplemented by 10% fetal calf serum (FCS, #A4766801, Gibco^TM^), 1% penicillin-streptomycin (P/S, #15240-62, Gibco^TM^), and 0.05% gentamicin (#15710064, Gibco^TM^). When confluency reached 80%, MSCs were passaged once before use according to manufacturer’s instructions. Briefly, cells were rinsed with Dulbecco’s PhosphateBuffered Saline (D-PBS, #14190-144, Gibco^TM^) and detached with TrypLE^TM^ Express Dissociation Reagent (TrypLE, #12604-021, Invitrogen). TrypLE was neutralized by addition of MSC medium with 10% FCS, and MSCs were reseeded.

For OPN preconditioning, MSC culture medium was removed and replaced with full MSC culture medium containing 1000 ng/mL OPN (#O2260, Sigma-Aldrich, Merck KGaA, Darmstadt, Germany) or control medium (0 ng/mL OPN) for 24 h. The concentration of 1000 ng/mL OPN was selected based on a dose-response experiment (of 200 to 5000 ng/mL OPN) showing maximal enhancement of MSC migration, in line with previous studies [44,45,46,48]. MSCs were collected immediately after preconditioning for experimental use.

### 2.3. Membrane Protein Extraction from MSCs

MSCs were plated in a 6-well plate at 0.2 × 10^6^ cells/well. After 4 days, wells were washed with ice-cold dPBS, and cells were harvested on ice with a cell scraper in 500 µL/well of cell fraction lysis buffer containing 20 mM Tris-Hcl (#10708976001, Sigma-Aldrich and #1003171000, Suppelco, Sigma-Aldrich), 2 mM EDTA (pH 8.0, #798681-1 kg, Merck KGaA), and 1 tablet of EDTA-free Pierce^TM^ Protease Inhibitor Mini Tablets (#A32955, Thermo Fisher Scientific). Cell lysate was collected, sonicated for 5 s on ice, and spun down at 500× *g* for 5 min at 4°C. Supernatant was transferred into precooled tubes and further spun down at 32,000 rpm in an ultracentrifuge (OPTIMA TLX9702, CTX97H02, Beckman, Brea, CA, USA) for 20 min at 4°C, and the supernatant was stored as the cytosol fraction at −20°C. Next, 50 µL of pellet lysis buffer, which contained 20 mM Tris-HCl, 2 mM EDTA at pH 8.0, 1% Triton X-100 (#1.086.031.000, VWR, Radnor, PA, USA), and 1 tablet of Mini EDTA-free protease inhibitor, was added to the pellet. Pellets were resolved by sonication at 3 × 5 s on ice and stored as plasma membrane fraction at −20°C until use in Western blot for OPN receptor cluster of differentiation 44 (CD44) and integrin β1 (Itgβ1). Protein levels were quantified with the Bradford protein assay (500-0006, Bio-Rad, Hercules, CA, USA) following the manufacturer’s instructions with BSA (#A3059-100 G, Sigma-Aldrich) used as a standard for the reference curve. Absorbance was measured at 595 nm using SKanIt^TM^ (Thermo Fisher Scientific). The linear regression coefficient (R^2^) of the standard curve was >0.98.

### 2.4. Total Protein Extraction from MSCs

MSCs were plated in a 6-well plate at 0.4 × 10^6^ cells/well. The day after, MSC medium was replaced with fresh culture medium containing 0 ng/mL or 1000 ng/mL of OPN for 1 h. Subsequently, wells were washed with ice-cold D-PBS, and cells were harvested on ice with a cell scraper in lysis buffer containing RIPA (#0278, Sigma-Aldrich), 1 tablet of Mini EDTA-free protease inhibitor, and phosphatase inhibitors sodium fluoride (NaF, 5 mM, #1.064.490.250, VWR) and sodium orthovanadate (Na_3_VO_4_, 1 mM, #81104.36, VWR). Cell lysates were collected, sonicated for 5 s on ice, and spun down at 17,000× *g* for 15 min at 4°C. Supernatant was stored at −20°C until use in Western blot for (phosphorylated) mitogen-activated protein kinase (MAPK/ERK 1/2). Protein levels were quantified with the Bradford protein assay as described above.

### 2.5. Western Blot

A total of 15 μg of protein was loaded for each sample onto a 4–20% Criterion^TM^ TGX^TM^ Precast Midi Protein Gel (#5671093, Bio-Rad) submerged in running buffer consisting of 1.44% glycine (#G8898, Sigma-Aldrich), 1% SDS (#1610418, Sigma-Aldrich), and 0.3% TRIS in dH_2_O. Electrophoresis ran for 1 h at 180 V and 400 mA. Separated proteins were transferred to nitrocellulose membranes using transfer buffer containing 1.4% glycine and 0.3% Tris in dH_2_O cooled with ice packs for 1 h at 100 V. Afterwards, membranes were washed in washing buffer containing Tween-20 (1:1000, #P9416, Sigma-Aldrich) and 0.1 M Tris at pH 7.5 in dH_2_O. Next, membranes were washed and blocked for 1 h in washing buffer with 5% skim milk powder (#79166-500 g, Merck KGaA) for CD44 and Itgβ1 or with 5% bovine serum albumin (BSA) for (p)ERK. After blocking, membranes were incubated overnight at 4°C on a mild shaker with primary antibody rabbit-anti-CD44 (1:1000, #37259, Cell Signaling Technology, Denver, MA, USA), rabbit-anti-Itgβ1 (1:1000, #34971, Cell Signaling Technology), or rabbit-anti-MAPK/ERK 1/2 (1:1000, #4695S, Cell Signaling Technology) diluted in blocking buffer. The following day, membranes were washed and incubated with secondary antibody HRP-linked-anti-rabbit IgG (1:5000, #NA934V, GE Healthcare, Little Chalfont, UK) for 1 h at RT. Imaging was performed using enhanced chemiluminescence (ECL, #RPN2106, Thermo Fisher Scientific) according to the manufacturer’s instructions with a Amersham IQ800 Imager (Cytiva Life Sciences, Marlborough, MA, USA). After imaging, membranes were stripped using sodium-azide (1:1000, #S2002-100 G, Sigma-Aldrich) in washing buffer for 20 min at RT, followed by incubation with 100 mM glycine (#8160130250, Sigma-Aldrich) at pH 2.0. Thereafter membranes were treated as described above, first with blocking buffer and then with primary antibody rabbit-anti-phosphoMAPK (pERK 1/2, 1:2000, #4370s, Cell Signaling Technology) and with secondary antibody HRP-linked-anti-rabbit IgG, followed by imaging. Mean gray values of pERK expression for each sample were analyzed in Fiji and normalized by their non-phosphorylated counterpart ERK.

### 2.6. Transwell Migration Assay

Migration of MSCs towards 10% FCS was assessed in vitro with a transwell migration assay. 0.25 × 10^5^ MSCs were resuspended in 100 μL of serum-free DMEM:F12 and loaded in the upper part of a transwell membrane insert (8 μm pore size, #CLS3422, Merck KGaA) hanging in a 24-well plate (#3524, Corning). To the lower wells, 600 μL of DMEM:F12 was added, containing 0% FCS or 10% FCS. MSCs were allowed to migrate in the incubator for 6 h and were thereafter fixed by submerging the inserts in ice-cold methanol (#322122, Sigma-Aldrich) for 10 min. Non-migrated cells were gently removed with a cotton tip from the upper side of the membrane. Cells that migrated to the bottom side of the membrane were stained with 4′,6-diamidino-2-phenylindole (DAPI, 288 nM, #D9542, Merck KGaA) for 5 min at RT. After being washed in dH_2_O, membranes were detached from the insert, mounted cell side up on Superfrost Plus slides (#6310108, VWR) with one drop of Fluorsave (#345789, VWR), and covered with high-precision, 24 × 50 mm #1.5H coverslips (#630-2187, VWR). Four pictures at distinct locations were taken per membrane at 20×/0.4NA magnification using an Axio Observer Z1 Microscope equipped with AxioCam MRm and ZEN software v1.1.1.0. (Carl Zeiss, Oberkochen, Germany). The number of DAPI^+^ cells was counted in Fiji v1.54 using the Analyze Particle plugin.

### 2.7. MSC Gene Expression

MSCs were cultured and seeded at a concentration of 0.2 × 10^5^ cells per well in 6-well plates. After 24 h, MSCs were preconditioned with 0 ng/mL or 1000 ng/mL of OPN in MSC medium for 24 h. Upon preconditioning, wells were washed with ice-cold D-PBS, and cells were lysed in RLT buffer (#79216, Qiagen, Hilden, Germany) with β-mercaptoethanol (1:1000, #161-0710, Bio-rad), aliquoted, and stored at −80 °C. RNA was isolated from MSC lysates using the RNeasy mini kit (#74104, Qiagen) combined with RNase-Free DNase Set (#79254, Qiagen) for DNase digestion, according to the manufacturer’s instructions. RNA quantity was assessed using the Nanodrop 2000 (Thermo Scientific), and quality on OD 260/280 ratio was between 1.97 and 2.3. Subsequently, 0.5 µg of RNA was synthesized to cDNA using the iScript reverse transcription supermix (#1708840, Bio-rad) according to the manufacturer’s instructions on a T100 Thermal Cycler (Biorad). The expression of brain-derived neurotrophic factor (*Bdnf*), cyclooxygenase-2 (*Cox-2*), fibroblast growth factor 2 (*Fgf-2*), interleukin 6 (*Il-6*), osteopontin (*Opn*), nerve growth factor (*Ngf*), transforming growth factor beta (*Tgf-β*), and vascular endothelial growth factor (*Vegf*) (see Table 1 for primer sequences) was measured by quantitative reverse transcription (qRT)-PCR (QuantStudio 3, Thermo Fisher Scientific) on the synthesized cDNA. All data were normalized for expression of β-actin, with the ΔΔCt method being used to obtain a fold change over non-preconditioned samples.

### 2.8. TGF-β ELISA

MSCs were cultured and seeded at a concentration of 0.2 × 10^5^ cells per well in 6-well plates. After 24 h, MSCs were preconditioned with 0 ng/mL or 1000 ng/mL of OPN in MSC medium for 24 h. Upon preconditioning, total mouse TGF-β1 was measured using an ELISA kit (#BMS608-4, Thermo Scientific) according to the manufacturer’s instructions. Briefly, 20 µL of samples was prepared diluted with 180 µL of assay buffer, followed by an incubation with 20 µL of 1 N HCl for 1 h at RT. Samples were neutralized with 20 µL of 1 N NaOH and vortexed. After the pre-coated wells were washed, 100 µL of samples were added for 2 h at RT. Wells were washed and subsequently treated with 100 µL of biotin conjugate for 1 h at RT. After washing, wells were incubated with 100 µL of streptavidin-HRP for 30 min on a microplate shaker at RT. After wells were washed, 100 µL of TMB substrate was added, and the plate was incubated protected from light for 30 min at RT. Lastly, the reaction was stopped by addition of 100 µL of stop solution, and the plate was read at 450 nm.

### 2.9. Primary Microglia Culture

Primary cultures of isolated cortical microglia were prepared from P1 C57BL/6 mice. Pups were killed by overdose of pentobarbital followed by decapitation. Brains were quickly dissected from the skull and submerged in dissection medium (Gey’s balanced salt solution (GBSS, #G9779-500 mL, Merck KGaA) containing 1% P/S and 30 mM D-(+)-of glucose (#G7021, Merck KGaA). First, meninges were removed, next the cerebellum and bulbi were removed, and then both cortices were dissected. Cortices were manually minced with a scalpel and incubated with 0.25% trypsin (#T4799, Sigma-Aldrich) in dissection medium for 15 min at 37 °C. The dissociated cell suspension was resuspended until homogenous and cultured in PLO-coated T75 flasks in glia culture medium containing DMEM/Ham:F10 (1:1, #41965-039 and #31550-023, Gibco^TM^) supplemented with 10% FCS, 2 mM glutamine (#25030024, Invitrogen), and 1% P/S. The cell suspension was diluted to plate cells of 1 animal (2 cortices) per T75 flask. The next day, the culture medium was refreshed. After 10–12 days in vitro, culture flasks were shaken for 20–22 h at 130–135 rpm at 37 °C to detach microglia. The microglia were collected by centrifugation at 300× *g* for 10 min at RT, counted, and seeded in PLO-coated 24-well plates at a density of 0.15 × 10^6^ cells/well. Microglia were cultured for 24 h before the non-contact MSC-microglia co-culture was started. After shaking, flasks were directly replenished with new culture medium, and new microglia were harvested with a second shake after another 10 days in vitro. Primary microglia were isolated on two separate days (Control: *n* = 6; LPS/-MSC: *n* = 6; LPS/naïve-MSC: *n* = 6; LPS/OPN-MSC: *n* = 6 on each isolation day).

### 2.10. Non-Contact MSC/Microglia Co-Culture

MSCs were preconditioned with 0 ng/mL or 1000 ng/mL of OPN 24 h prior to the start of non-contact co-culture with primary microglia. Naïve- or OPN-MSCs were harvested and embedded at a density of 0.4 × 10^6^ MSCs in 75 µL of TrueGel 3D Hydrogel (#TRUE1, Sigma-Aldrich) per transwell insert (Millicell^®^ 24 well Hanging Cell Culture Inserts, PET 0.40 µm, #PTHT24H48, Millipore, Merck KGaA) according to the manufacturer’s instructions. Embedded MSCs were allowed to equilibrate for 3 h. Thereafter, microglia were stimulated with 50 ng/mL of lipopolysaccharide (LPS from Escherichia coli O127:B8, #L4515, Merck KGaA), and the transwell inserts containing naïve- or OPN-MSCs were added to each well to start the co-culture. After 24 h, inserts were removed, and microglia supernatant was collected, aliquoted, and stored at −80 °C for tumor necrosis factor alpha (TNF-α) analysis by ELISA.

### 2.11. TNF-α ELISA

TNF-α concentration in the microglia supernatant was measured using an ELISA kit for murine TNF-α (#CT303A, Ucytech, Utrecht, The Netherlands) according to the manufacturer’s instructions. Briefly, wells were coated overnight at 4 °C with coating antibody (1:100) in PBS. The next day, wells were washed with PBS containing 0.05% Tween-20 and incubated for 1 h at RT with blocking buffer consisting of 1% BSA in PBS. Blocking buffer was removed and the microglia supernatant samples (diluted 1:800 in PBS containing 0.5% BSA and 0.05% Tween-20) were incubated for 2 h at RT. Next, wells were washed in 0.05% Tween-20/PBS, followed by incubation with detection antibody (1:100 in 0.5%BSA/0.005%Tween-20/PBS) for 1 h at RT. After washing, 100 µL of SPP conjugate (1:100 in 0.5%BSA/0.005%Tween-20/PBS) was added to each well and incubated in the dark for 1 h at RT. Afterwards, wells were washed, ready-to-use TMB substrate solution (#CT303A, Ucytech) was added, and the plate was incubated protected from light for 20 min at RT. Lastly, the reaction was stopped by addition of 100 µL of ready-to-use stop solution (#CT303A, Ucytech), and the plate was read at 450 nm.

### 2.12. Neural Stem Cell Culture

Cortical stem cells (NSCs, #SCR029, Sigma-Aldrich) from embryonic day 15–18 C57BL/6 mice were cultured as neurospheres in 6-well plates (#3516, Corning) at a density of 0.2 × 10^6^ cells/well in NSC medium consisting of DMEM:F12 supplemented by 2% B27 without vitamin A (#12587001, Gibco^TM^) and 1% P/S. Daily, 20 ng/mL of Recombinant Human Fibroblast Growth Factor-basic (bFGF, #100-18B, Peprotech, Rocky Hill, NJ, USA) and 20 ng/mL of Recombinant Human Heparin Binding Epidermal Growth Factor (EGF, #100-47b, Peprotech) were supplied to the NSCs to induce proliferation and retain stemness. NSCs were cultured according to the manufacturer’s protocol and used at P3. Briefly, neurospheres were collected for passaging, centrifuged at 300× *g* for 5 min, and disrupted by pipetting. Subsequently, cells were counted on a NC-200 (ChemoMetec, Allerod, Denmark) and subcultured in 6-well plates at a density of 0.2 × 10^6^ viable cells per well.

### 2.13. Non-Contact MSC/NSC Co-Culture

NSCs were plated at a density of 0.4 × 10^5^ cells per well in 24-well plates coated with 10 µg/mL of poly-L-ornithine (PLO, #P3655, Merck KGaA) and 5 µg/mL of laminin (#L2020, Merck KGaA) and cultured in the presence of 20 ng/mL of bFGF and of EGF to retain stemness. At 24 h after preconditioning with 0 ng/mL or 1000 ng/mL OPN, naïve- or OPN-MSCs were harvested and embedded at a density of 0.8 × 10^5^ MSCs per 75 µL of TrueGel 3D Hydrogel per transwell insert according to manufacturer’s instructions. The medium in the inserts was changed to MSC medium containing 2 U/mL of Heparin Sodium Injection (#H3149-10KU, Merck KGaA) and 5% Platelet Lysate (PL, Lonza, Walkersville, MD, USA) instead of standard MSC culture medium since NSCs in the bottom well failed to grow in FCS-containing medium in the inserts. Embedded MSCs were allowed to equilibrate for 3 h. NSC differentiation was initiated by removal of bFGF and EGF, and co-culture was started by transferring the transwell inserts containing naïve- or OPN-MSCs into the NSC wells. Inserts were refreshed daily to maintain the preconditioned state of the OPN-MSC condition. As the control condition, NSCs were differentiated in co-culture with gel inserts without MSCs (i.e., empty inserts). The assay was stopped at 72 h by withdrawing MSC inserts and fixing differentiated NSCs with 4% PFA in D-PBS.

### 2.14. Immunocytochemistry

Fixed NSC cultures were washed with PBS and blocked with 2% BSA and 1% saponin for 25 min. Subsequently, the plates were incubated with primary antibody rabbit anti-β-III-tubulin (βIII-tubulin (βIIIT), 1:1000, #Ab18207, Abcam, Cambridge, UK) for 1 h at RT and washed with PBS, followed by incubation with conjugated secondary antibody anti-rabbit Alexafluor-594 (1:250, Invitrogen) for 1 h at RT. Cell nuclei were counterstained with DAPI and embedded in FluorSave. Fluorescent images (*n* = 5 per well) were acquired at 10×/0.3 NA magnification using an Axio Observer Z1 Microscope equipped with AxioCam MRm and ZEN software. Fiji v1.54 was used to quantify β-III-tubulin^+^ area and to count the number of DAPI^+^ cells. For each well, values of all acquired pictures were first normalized by DAPI count, averaged, and then normalized to average value of the negative control condition (empty inserts). To measure neuronal complexity, βIII-tubulin^+^ neurons (minimum *n* = 15 per well) were analyzed using the autopath filament tracer algorithm of Imaris v9.2. βIII-tubulin^+^ neurons were manually traced, neuronal complexity was assessed by total neurite length and by the number of branch points, segments, terminal points, and Sholl intersections. Neurons were excluded if branches were obscured by nearby cells or by background staining or if neurites were incomplete.

### 2.15. Animals and HI Injury Model in Neonatal Mice

C57Bl/6 mice (OlaHsa, ENVIGO, Horst, The Netherlands) were kept in individually ventilated cages with woodchip bedding, cardboard shelters, and tissues provided. Animals were kept on a 12 h day/night cycle in a temperature-controlled room at 20–24 °C and 45–65% humidity with ad libitum food and water access. Mice were bred in-house by placing males and females together in a ratio of 1:1 or 1:2 for 5 days, and afterwards dams were housed solitarily to give birth. Day of birth was considered as postnatal day (P)0. Hypoxic-ischemic (HI) injury was induced in P9 pups weighing at least 3.0 g by permanent unilateral right carotid artery ligation under isoflurane anesthesia (5–10 min; 5% induction, 3–4% maintenance with flow O2: air 1:1), followed by recovery with their mother for at least 75 min. Subsequently, pups were exposed to systemic hypoxia at 10% O_2_ for 45 min in a temperature-controlled and humidified hypoxic incubator. Xylocaine (#N01BB02, AstraZeneca, Cambridge, UK) and bupivacaine (#N01BB01, Actavis, Allergan Inc, Dublin, Ireland) were applied to the wound for pre- and post-operative analgesia, respectively. Control sham-operated (SHAM) animals were subjected to anesthesia and surgical incision only, without ligation or exposure to hypoxia. A total of 81 pups underwent a successful HI procedure (mortality during surgery was of 5/86 animals = 5.8%).

### 2.16. Brain Gene Expression Profiling

To determine growth factor gene expression in the brain, SHAM-control and HI pups were euthanized at P12 (i.e., 3 days after induction of HI) by overdose of pentobarbital, followed by decapitation, and collected hemispheres were rapidly frozen in liquid nitrogen and stored at −80°C until use. Frozen hemispheres (SHAM: *n* = 7; HI: *n* = 9) were crushed on liquid nitrogen, and RNA was isolated with the RNeasy mini kit (Qiagen). RNA quantity and quality were assessed by spectrophotometry at 260 mm, and OD 260/280 ratio was determined to evaluate quality. cDNA transcription was performed using the RT2 first-strand synthesis kit (Qiagen) according to the manufacturer’s protocol. Per each condition, cDNA samples were pooled and growth factors’ mRNA expression levels were determined by RT^2^ Profiler™ PCR Array Mouse Growth factors PAMM-041ZA (#330231, Qiagen) on the CFX96 real-time PCR detection system (Biorad, Hercules, CA, USA) according to the manufacturer’s instructions. Data were normalized using multiple housekeeping genes (Gapdh and Hsp90ab1) provided within the PCR array and analyzed by comparing 2^−ΔΔCt^ using Qiagen software. Gene expression changes in the ipsilateral hemisphere upon HI were calculated relative to the contralateral hemisphere or to the ipsilateral hemisphere of SHAM brains. A fold regulation threshold of 2.0 was considered as either down- or upregulation.

### 2.17. Intranasal Mesenchymal Stem Cell Treatment

Male and female offspring that survived 2 days after induction of HI were included in the efficacy study and were randomly assigned to the 4 experimental groups, with an intended 50/50 ratio between female and males (Table 2). Group sizes were determined based on effect size of naïve-MSC treatment in previous experiments using the same techniques by performing power analysis. During the course of study, the mortality rate was 4.2% (3 out of 72 HI-injured animals). Three days after induction of HI, MSCs at passage 10 were administered intranasally to mouse pups. Hyaluronidase (100U, #H4272, Sigma-Aldrich) was administered intranasally 30 min before MSC administration to increase the permeability of the connective tissue in the nasal cavity (3 × 2 µL per nostril; 12 µL in total). HI-injured mouse pups were treated intranasally with naïve-MSCs or OPN-preconditioned (OPN-) MSCs (0.5 × 10^6^ per animal) in D-PB, or in D-PBS as vehicle (VEH) solution by administration of 3 droplets of 2 μL per nostril (12 µL in total).

### 2.18. Histology, Image Acquisition, and Analysis of Brain Tissue

At 28 days after induction of HI (P37), animals were euthanized by overdose of pentobarbital (Alfasan, Woerden, The Netherlands), followed by transcardial perfusion with phosphate-buffered saline (PBS, #524650-1, VWR) and 4% paraformaldehyde (PFA, #VWRK4078-9020, VWR). Brains were collected, post-fixed for 24 h in 4% PFA, dehydrated in increasing ethanol concentrations to 100%, and afterwards embedded in paraffin. Coronal sections of 8 µm were cut at the level of the hippocampus (bregma level −1.85 mm in adult brain). Subsequently, sections were deparaffinized and rehydrated. For assessment of ipsilateral tissue loss, sections were stained with hematoxylin (#517-28-2, VWR) for 5 min and eosin (#4082-9002, Klinipath, Duiven, The Netherlands) for 2 min (H&E staining). Next, sections were dehydrated in increasing ethanol concentrations and mounted in DEPEX (#18243.01, Brunschwig, Amsterdam, The Netherlands). Full-section images were captured with a Nikon-D1 camera (Nikon, Tokyo, Japan), and the H&E^+^ area of the ipsilateral and contralateral hemisphere was assessed using Fiji v1.54d (National Institute of Health, NIH, Bethesda, MD, USA). Ipsilateral tissue loss was calculated as [(1 − ipsilateral hemisphere area/contralateral hemisphere area) × 100%].

### 2.19. Statistical Analysis

In the in vivo and in vitro analyses, the experimental units were individual mice or individual wells, respectively. Statistical analysis and visualization of data were performed by using GraphPad Prism v10.4.0 (Boston, MA, USA). First, significant outliers were detected by the ROUT outlier method (Q = 1%), and then data were checked for normal Gaussian distribution. Unpaired *t*-test (2 groups) or one- or two-way ANOVA followed by uncorrected Fisher’s LSD post hoc tests (>2 groups) were performed for group comparisons. Statistical tests used are reported in the figure legends. Raw data are shown as the mean ± standard error of the mean (SEM). Results were considered statistically significant if *p* < 0.05.

## 3. Results

### 3.1. Osteopontin Preconditioning of MSCs Activates the Intracellular ERK Pathway, Enhances Their Migratory Capacity, and Changes MSC Gene Expression

**Figure 1:**
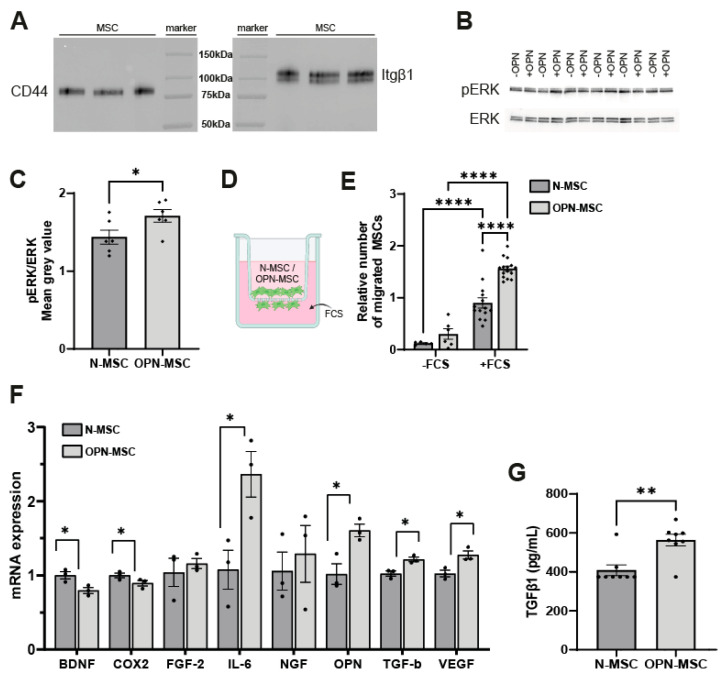
MSCs express osteopontin receptors, and osteopontin preconditioning of MSCs activates the ERK pathway, enhances MSC migratory capacity in vitro, and changes gene expression in MSCs. (**A**) Western blot showing CD44 (left) and Itgb1 (right) protein expression in the plasma membrane of MSCs. (**B**) Western blot showing phospho-ERK and total ERK protein expression in naïve-MSCs and OPN-MSCs. (**C**) Quantification of phospho-ERK normalized by total ERK protein expression (*n* = 6 wells per condition). (**D**) Schematic overview of transwell migration assay with MSCs in the hanging insert and addition of 10% FCS in the lower chamber to induce MSC migration. (**E**) Quantification of numbers of N-MSCs or OPN-MSCs migrated towards 10% FCS relative to the +FCS/N-MSC condition. -FCS: *n* = 5–6 wells per condition; +FCS: *n* = 14–15 wells per condition out of 2 independent experiments. (**F**) Quantification of brain-derived neurotrophic factor (*Bdnf*), cyclo-oxygenase 2 (*Cox2*), fibroblast growth factor 2 (*Fgf-2*), interleukin 6 (*Il-6*), nerve growth factor (*Ngf*), osteopontin (*Opn*), transforming growth factor β (*Tgf-β*), and vascular endothelial growth factor (*Vegf*) mRNA expression by qPCR in MSCs immediately after preconditioning with 0 ng/mL or 1000 ng/mL of OPN for 24 h. *n* = 3 wells per condition. (**G**) Quantification of TGF-β total protein secreted by MSCs after preconditioning with 0 ng/mL or 1000 ng/mL of OPN for 24 h. *n* = 8 wells per condition. All data represent mean ± SEM. Statistics were performed by unpaired *t*-test (**C**,**F**), Mann-Whitney test (**G**), and one-way ANOVA followed by uncorrected Fisher’s LSD post hoc tests (**E**). * *p* < 0.05, ** *p* < 0.01, **** *p* < 0.0001 between indicated groups. ERK: extracellular-signal-regulated kinase; FCS: fetal calf serum; Itgβ1: integrin subunit β 1; N-MSC: naïve mesenchymal stem cells; OPN-MSC: osteopontin-preconditioned mesenchymal stem cells.

First, we confirmed by Western blot that MSCs express OPN receptors CD44 and Itgβ1 on their plasma membrane: i.e., positive bands at 80 kDa (CD44) and 120 kDa (Itgβ1) were observed (Figure 1A). To confirm that MSCs respond to OPN preconditioning by intracellular cascades, activation of the extracellular=signal-regulated kinases (ERK) signaling pathway was assessed by Western blot analysis. Preconditioning of MSCs with 1000 ng/mL of OPN for 1 h led to significant increased phosphorylation of ERK compared to levels in naïve-MSCs (*p* = 0.0450, Figure 1B,C). Next, we examined whether OPN preconditioning for 24 h could affect the migratory capacity of MSCs in vitro by using a transwell migration assay (Figure 1D). Both naïve-MSCs and OPN-MSCs migrated in significantly higher numbers to 10% FCS compared to no FCS (*p* < 0.0001, Figure 1E). Interestingly, OPN preconditioning significantly increased the number of migrated MSCs to FCS compared to no preconditioning (*p* < 0.0001).

Next, we investigated whether OPN preconditioning of MSCs alters the gene expression of selected cytokines and the trophic and growth factors involved in neuroregeneration and immunomodulation (Figure 1G). Immediately following OPN preconditioning for 24 h, cyclooxygenase-2 (*Cox2*, *p* = 0.0492) and brain-derived neurotrophic factor (*Bdnf*, *p* = 0.0301) were downregulated in OPN-MSCs compared to naïve-MSCs, whereas interleukin-6 (*Il-6*, *p* = 0.0168), transforming growth factor-β (*Tgf-β*, *p* = 0.0144), and vascular endothelial growth factor (*Vegf*, *p* = 0.0231) were upregulated. No significant changes were found in mRNA expression of fibroblast growth factor 2 (*Fgf-2*, *p* = 0.5909) and nerve growth factor (*Ngf*, *p* = 0.6374). Interestingly, OPN preconditioning also led to upregulation of *Opn* in MSCs (*p* = 0.0217), suggesting an autocrine feedforward loop. To confirm that OPN preconditioning alters the MSC secretome, TGF-β protein was measured in MSC supernatants. OPN preconditioning significantly increased the levels of TGF-β secretion by MSCs (*p* = 0.0079, Figure 1G).

Together, these data support that MSCs are responsive to OPN exposure as illustrated by activation of the ERK pathway in MSCs, and OPN preconditioning enhances their migratory capacity and leads to transcriptional regulation of multiple factors in MSCs that have neurotrophic and immunomodulatory properties.

### 3.2. Osteopontin Preconditioning Does Not Impair Anti-Inflammatory Capacity of MSCs

**Figure 2:**
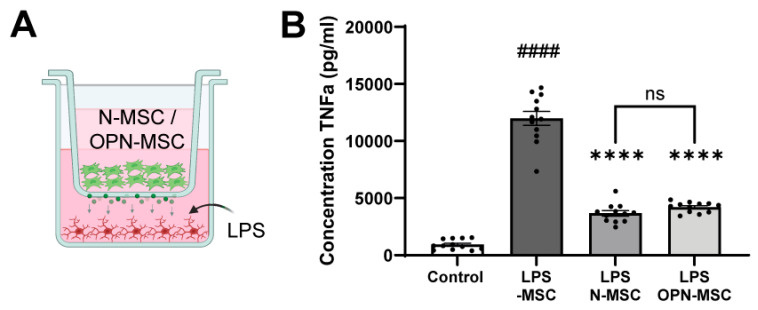
OPN-MSCs are equally effective to naïve-MSCs in reducing TNF-α secretion by microglia after LPS exposure in vitro. (**A**) Schematic overview of non-contact co-culture between naïve-MSCs or OPN-MSCs in the hanging insert and primary-isolated mouse microglia exposed to 50 ng/mL of LPS in the lower compartment. The co-culture allowed the MSC secretome (green dots) to reach the microglia compartment. (**B**) Quantification of tumor necrosis factor α (TNF-α) secretion by microglia after 24 h of LPS exposure (All groups: *n* = 13–18 wells per condition from 3 different shakes). Data represent mean ± SEM. Statistics were performed by one-way ANOVA followed by uncorrected Fisher’s LSD post hoc tests: #### *p* < 0.0001 compared to no LPS exposure; **** *p* < 0.0001 compared to the +LPS/-MSC condition; ns: non-significant between indicated groups. LPS: lipopolysaccharide; N-MSC: naïve mesenchymal stem cells; OPN-MSC: osteopontin-preconditioned mesenchymal stem cells.

Due to the increased expression of *Il-6*, which could indicate MSCs might lose some of their potent anti-inflammatory capacity after OPN preconditioning, we performed a non-contact co-culture between MSCs and LPS-stimulated primary microglia (Figure 2A). Exposure of microglia to LPS strongly increased the secretion of pro-inflammatory tumor necrosis factor-α (TNF-α) (*p* < 0.0001 compared to non-stimulated microglia, Figure 2B). Co-culture with both naïve-MSCs and OPN-MSCs led to a significant decrease in TNF-α secretion by LPS-exposed microglia compared to those not exposed to MSCs (LPS/naïve-MSC versus LPS/-MSC: *p* < 0.0001; LPS/OPN-MSC versus LPS/-MSC: *p* = 0.0004). No significant differences in the dampening of TNF-α secretion by LPS-stimulated microglia were detected between naïve-MSCs and OPN-MSCs (*p* = 0.7019), indicating that the strong anti-inflammatory capacity of MSCs is retained following OPN preconditioning.

### 3.3. Osteopontin Preconditioning of MSCs Enhances Their Neurotrophic Potential

**Figure 3:**
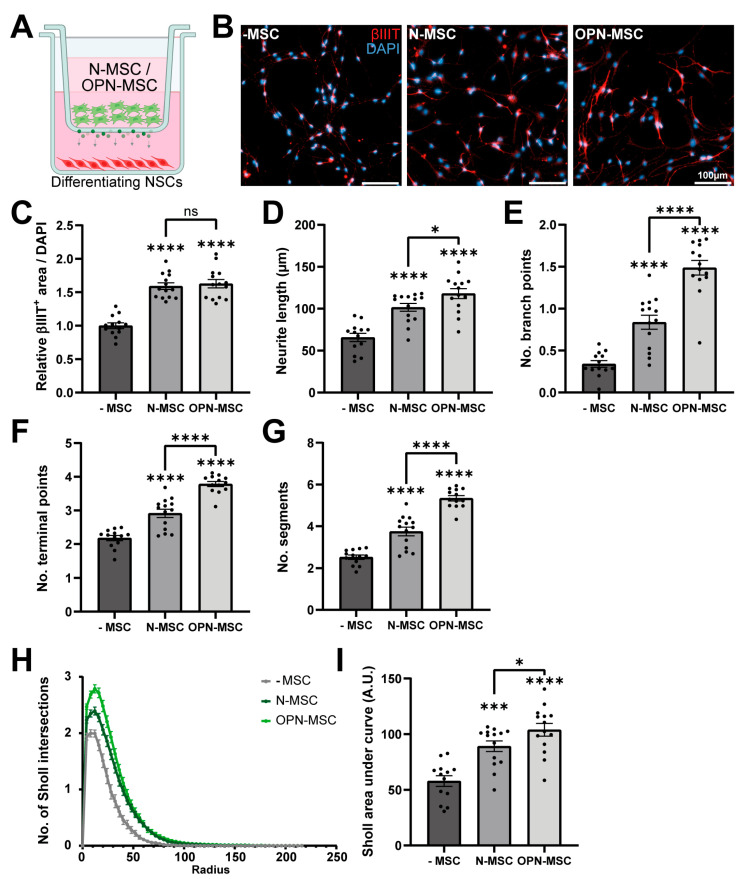
Osteopontin preconditioning enhances the neurotrophic potency of MSCs. (**A**) Schematic overview of non-contact co-culture of neural stem cells (NSCs) with naïve-MSCs or OPN-MSCs in the hanging insert, allowing the MSC secretome (green dots) to reach the NSCs compartment. (**B**) Representative fluorescent images (10×/0.3 NA) of βIII-tubulin (βIIIT)^+^ neurons. (**C**) Quantification of βIIIT^+^ area normalized to the number of DAPI^+^ nuclei and relative to the -MSC (empty insert) condition. Quantifications of (**D**) neurite length, (**E**) number of branch points, (**F**) terminal points, and (**G**) segments of βIIIT^+^ neurons as measures of neuronal complexity. (**H**) Number of intersection of βIIIT^+^ neurons with circles of increasing radius in Sholl analysis. (**I**) Quantification of Sholl analysis by area under the curve. Data represent mean ± SEM. Statistics were performed by one-way ANOVA followed by uncorrected Fisher’s LSD post hoc tests (C-G,I): * *p* < 0.05, *** *p* < 0.001, **** *p* < 0.0001 relative to the -MSC condition or between indicated groups. (C-G,I): *n* = 13–14 wells per condition out of 2 independent experiments. ns: non-significant between indicated groups. -MSC: no mesenchymal stem cells (empty insert); N-MSC: naïve mesenchymal stem cells; OPN-MSC: osteopontin-preconditioned mesenchymal stem cells.

Next, we assessed the effect of OPN preconditioning on the neurotrophic properties of MSCs in vitro in a non-contact co-culture of MSCs with differentiating differentiated neural stem cells (NSCs) (Figure 3A). Differentiated NSCs co-cultured with naïve-MSCs or OPN-MSCs showed a higher βIII-tubulin^+^ area compared to NSCs differentiated in absence of MSCs (naïve-MSC vs. -MSC: *p* < 0.0001; OPN-MSC vs. -MSC: *p* < 0.0001, Figure 3B,C), indicating that MSCs promote neurogenesis. No differences were found in βIII-tubulin^+^ area between co-culture of NSCs with naïve-MSCs or that of NSCs with OPN-MSCs (*p* = 0.6144), suggesting that OPN preconditioning of MSCs does not influence their capacity to induce neuronal lineage commitment. Morphometric analysis of the βIII-tubulin^+^ neurons showed that co-culture with naïve-MSCs induced the formation of more complex neurons compared to no coculture (i.e., with empty [no MSCs] inserts), as indicated by longer neurites (*p* < 0.0001, Figure 3D) and higher numbers of branch points (*p* < 0.0001, Figure 3E), neurite terminal points (*p* < 0.0001, Figure 3F), and segments (*p* < 0.0001, Figure 3G), which resulted in higher overall branch complexity (*p* = 0.0001, Figure 3H,I). Importantly, OPN preconditioning further enhanced the neurotrophic potency of MSCs, as all morphometric parameters analyzed in differentiated neurons were increased when in co-culture with OPN-MSCs compared to in co-culture with naïve-MSCs (OPN-MSC vs. naïve-MSC Figure 3D: *p* = 0.0309; Figure 3E–G: *p* < 0.0001; Figure 3I: *p* = 0.0464). These data indicate that OPN preconditioning promotes the neurotrophic effects of MSCs and may therefore enhance their therapeutic efficacy in vivo.

### 3.4. Osteopontin Preconditioning Does Not Enhance the Therapeutic Efficacy of Intranasal MSC Therapy After Neonatal HI

**Figure 4:**
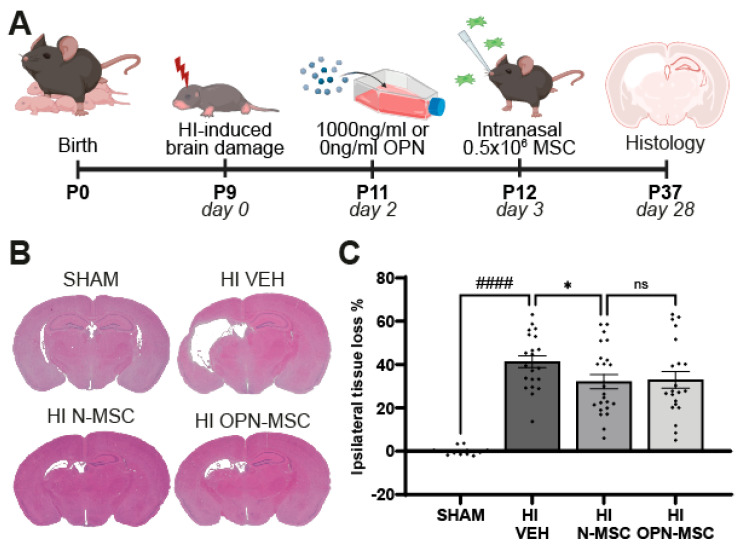
Osteopontin preconditioning of MSCs does not increase their therapeutic potential to reduce lesion size in a mouse model of neonatal HI brain injury. (**A**) Overview of study design. (**B**) Representative images of ipsilateral tissue loss at 28 days post-HI visualized by HE staining in SHAM-control mice or HI-injured mice intranasally treated with vehicle, naïve MSCs, or OPN-MSCs at 3 days after HI. (**C**) Quantification of ipsilateral tissue loss (%). SHAM: *n* = 13; HI VEH: *n* = 23; HI N-MSC: *n* = 24; HI OPN-MSC: *n* = 22 animals. Data represent mean ± SEM. Statistics were performed by one-way ANOVA followed by uncorrected Fisher’s LSD post hoc tests: ns: non-significant; * *p* < 0.05, #### *p* < 0.0001 between indicated groups. HI: hypoxia-ischemia; N-MSC: naïve mesenchymal stem cells; OPN-MSC: osteopontin-preconditioned mesenchymal stem cells; P: postnatal day; VEH: vehicle.

To investigate if OPN preconditioning enhances the therapeutic efficacy of intranasal MSC therapy, 0.5 × 10^6^ of OPN-MSCs or naïve-MSCs or vehicle solution were given intranasally at 3 days following the induction of neonatal HI brain injury on P9 (Figure 4A). At 28 days after the induction of HI, vehicle-treated HI-injured mice showed a significant lesion indicated by a ~40% tissue loss of the ipsilateral hemisphere compared to SHAM-control mice (*p* < 0.0001, Figure 4B,C). Ipsilateral tissue loss was significantly reduced by intranasal naïve-MSCs compared to vehicle treatment (*p* = 0.0369). However, OPN-MSCs did not further reduce HI lesion size compared to naïve-MSCs (*p* = 0.8494), suggesting that OPN preconditioning does not enhance the therapeutic effect of intranasal MSC therapy at 3 days following HI in mice.

## 4. Discussion

This study was the first to evaluate the impact of OPN preconditioning on the migratory, anti-inflammatory, and neurotrophic properties of MSC, and to assess its potential as a strategy to optimize the therapeutic efficacy of intranasal MSC administration for neonatal HI brain injury.

In line with previous studies, we demonstrated that the OPN receptors Itgβ1 and CD44 are present on the MSC membrane, enabling OPN binding, and that MSCs respond to OPN preconditioning by activating ERK signaling pathway [39,40,41,42]. Activation of this pathway has recently been identified as a key mechanism through which OPN promotes MSC migration, both in vitro and in vivo in the context of wound healing and bone remodeling [44,45,46,48,49,50,51,52,53]. Notably, our findings show that OPN plays an important role in MSC migration also when used as a preconditioning agent since OPN-MSCs exhibited enhanced chemotactic responsiveness compared to non-preconditioned MSCs. Future studies should investigate the in vivo migration of intranasally administered OPN-MSC using available tracing techniques [9,18,54].

Besides ERK signaling and migration, binding of OPN to its receptors results in activation of other cellular cascades and consequently in the participation of OPN in processes such as cell adhesion and differentiation, as well as the secretion of factors involved in angiogenesis and immunomodulation [39,40,41,42], thus possibly influencing the immunomodulatory functions of MSCs. Indeed, MSCs have been shown to possess potent anti-inflammatory properties, both in vivo after brain injury in neonatal mic, and in vitro after stimulation of microglia with inflammatory agents such as LPS [54]. Accordingly, in our study, we observed that naïve-MSCs strongly reduced TNF-α secretion by microglia after exposure to LPS. OPN preconditioning, however, did not further improve or impair the immunomodulatory capacity of MSCs. Although limited to only a few inflammatory markers, our gene expression analysis seems to confirm these findings. We observed differential regulation of genes with both pro- and anti-inflammatory properties, i.e., *Il-6*, *Tgf*-*β*, and *Cox-2,* after OPN preconditioning of MSCs. Elevated *Tgf-β* expression levels and protein secretion by OPN-MSCs could potentially contribute to an anti-inflammatory microglial phenotype, as *Tgf-β* signaling has been shown to prevent excessive microglial reactivity in models of central nervous system disease [55]. However, the simultaneous increase in *Il-6* expression may counterbalance this effect by promoting microglial activation [56,57]. *Cox-2*, typically associated with the pro-inflammatory phase of injury and cell death in CNS trauma [58], was downregulated in OPN-MSCs, which could suggest a beneficial, immunosuppressive effect. Yet, *Cox-2* downregulation in MSCs has also been linked to immunostimulatory effects, indicating a dual role for this gene [59,60]. Altogether, our findings on activated microglia indicate that OPN-MSCs display anti-inflammatory properties comparable to those of naïve-MSCs. However, in this study, we assessed the anti-inflammatory properties of MSCs using a robust and relevant assay for HI brain injury, yet this was limited to a single cellular context. It therefore remains possible that future investigations involving additional immune populations may uncover altered anti-inflammatory properties of MSCs induced by OPN preconditioning.

In addition to immunomodulation, differential expression of growth factors may affect the neuroregenerative capacity of OPN-MSCs. OPN preconditioning resulted in the upregulation of *Tgf-β*, *Il-6*, *Opn*, and *Vegf* expression and the downregulation of *Bdnf* expression. While the observed reduction in expression of neurotrophic factor *Bdnf* may limit the neurogenic capacity of OPN-MSCs, the increased expression of *Vegf* may enhance angiogenesis and tissue remodeling, thus indirectly facilitating neural repair [61,62]. Furthermore, since TGF-β is known to promote neuroprotection and neurite outgrowth [63,64], increased secretion of TGF-β by OPN-MSCs may underlie the enhanced neurotrophic effects of the OPN-MSC secretome observed in vitro. *Il-6* and *Opn*, while traditionally associated with inflammation, have also been implicated in the regulation of neurogenesis [26,65]. We show here that OPN preconditioning enhanced the neuroregenerative capacity of the MSC secretome, as OPN-MSCs enhanced the neuronal complexity of differentiated NSCs in a non-contact co-culture. A previous study showed that hippocampal neurons cultured on an OPN substrate displayed a higher number of primary dendrites compared to neurons cultured on a laminin substrate, and this growth was mediated through OPN receptors ITGβ1 and CD44 [66]. As our study showed that OPN preconditioning of MSCs led to increased *Opn* mRNA expression, it is possible that secreted OPN contributed to the enhanced neurotrophic properties of OPN-MSCs. Since OPN preconditioning caused increased *Opn* expression levels, our findings suggest a possible self-sustaining mechanism responsible for enhancing OPN-driven cellular functions via an autocrine feedforward loop. Previous evidence implies that such a positive feedforward loop might be triggered by ERK pathway activation [67,68]. To confirm this hypothesis, follow-up studies inhibiting ERK signaling and assessing OPN expression and neurotrophic properties by OPN-MSCs over time are required. Additionally, VEGF, TGF-β, and IL-6 have all been shown to promote neurite outgrowth in cultured neurons [64,69,70], so their enhanced presence in the MSC secretome could therefore potentially have improved the morphological characteristics of newly differentiated neurons. A limitation of this study is that we assessed the effect of OPN preconditioning on the production of these factors mainly at the gene expression level in MSCs. Future studies should therefore include a more comprehensive protein-level analysis of the MSC secretome at various timepoints following OPN preconditioning. This will help determine whether and for how long OPN-MSCs sustain elevated secretion of key neurogenic factors and whether the enriched OPN-MSC secretome is sufficiently potent to promote the recruitment of neural stem cells from neurogenic niches to the lesion site and to support in situ NSC differentiation.

Despite the enhanced migratory and neurotrophic capacity of OPN-MSCs in vitro, our in vivo data indicate that the current OPN preconditioning protocol does not improve the therapeutic efficacy of intranasal MSCs when administered at 3 days post-HI in a neonatal mouse model. A limitation of this study is that our in vivo therapeutic assessment focused solely on anatomical outcome, without evaluating functional recovery. It therefore remains unanswered whether OPN-MSCs might impact functional outcomes, such as cognitive and motor functions, even in the absence of structural improvement. As discussed above, the results from the NSC differentiation assay suggest that OPN-MSCs, compared to naïve MSCs, promoted neuronal complexity but did not enhance the formation of new neurons as such. In contrast, our recent work showed that another preconditioning strategy, i.e., hypoxic preconditioning of MSCs, enhanced both the number and the complexity of newly differentiated neurons [18]. These findings suggest that improvements in both neurogenesis and neurotrophic support may be necessary to enhance therapeutic outcomes following HI. Future research should focus on identifying an optimal concentration of OPN and the timeframe for preconditioning that provides enhancement of neurogenic as well as neurotrophic properties of MSCs. Another important consideration in the failure of enhanced therapeutic potency of OPN-MSCs in vivo are the elevated levels of endogenous OPN in the brain as a response to injury [26]. Increased levels of OPN protein have been frequently observed in models of (neonatal) ischemic brain injury until at least 5 days post-insult [25,26,71,72,73,74,75]. We confirmed this in our neonatal HI model, demonstrating upregulated expression of several genes, including OPN, IGF-1, and S100A6 in the ischemic hemisphere at 3 days post-HI (Table S1). Particularly, OPN showed the most pronounced upregulation among the analyzed growth factors in the brain of HI-injured animals (194 fold increased). Given the timing of MSC administration in our study at 3 days post-HI and the fact that MSCs have been shown to reach the site of injury within 12 h after administration [9], it is safe to assume that MSCs, whether OPN preconditioned or not, were exposed to high OPN concentrations upon arrival in the injured areas. This could mean that once MSCs arrive at the lesion site, they would effectively undergo in situ OPN preconditioning, potentially explaining the lack of additional benefit of OPN-MSCs in reducing infarct size. Earlier studies proved a therapeutic window of intranasal MSC treatment of at least 10 days post-HI [9,10,11,12]. Future studies should explore whether delayed administration of OPN-preconditioned MSCs at day 10 post-HI, i.e., at a time point after injury when OPN in the lesion has declined [26], might reveal a therapeutic advantage of OPN preconditioning of MSCs.

## 5. Conclusions

In conclusion, despite the beneficial effects of OPN preconditioning on MSC migration and on their neurotrophic effects, our findings did not demonstrate enhanced therapeutic efficacy on brain injury of intranasal OPN-MSC therapy when administered 3 days post-HI in a neonatal mouse model. Nonetheless, our results highlight the potential of OPN preconditioning to optimize MSC mechanisms and underscore the need for further research to also assess functional outcomes and to determine whether OPN preconditioning may improve MSC potency at later time points after injury, when endogenous OPN levels have subsided.

## Figures and Tables

**Table 1:** Primers used for qPCR.

Name	Forward Sequence (5′ ⟶ 3′)	Reverse Sequence (3′ ⟶ 5′)
*Bdnf*	CACATTACCTTCCAGCATCTGTTG	ACCATAGTAAGGAAAAGGATGGTCAT
*Cox-2*	GGTCTGGTGCCTGGTCTG	CTCTCCTATGAGTATGAGTCTGC
*Fgf-2*	GCGAGAAGAGCGACCCACAC	GAAGCCAGCAGCCGTCCATC
*Il-6*	TCTAATTCATATCTTCAACCAAGAGG	TGGTCCTTAGCCACTCCTTC
*Opn*	TGGACTGAGGTCAAAGTCTAGGA	CCGCTCTTCATGTGAGAGGTGA
*Ngf*	ACGGGCAGCATGGTGGAG	TGTAGAACAACATGGACATTACGC
*Tgf-β1*	GTGACAGCAAAGATAACAAAC	CTGAAGCAATAGTTGGTATCC
*Vegf-b*	GATCCTCTGCCCGCCTTG	CCCGTGGAGTCTGGAAAGC
β-actin	AGAGGGAAATCGTGCGTGAC	CAATAGTGATGACCTGGCCGT

**Table 2:** Number of animals used for this study for gene expression and histology experiments. HI: hypoxia-ischemia; VEH: vehicle treatment; MSCs: mesenchymal stem cells; N-MSCs: naïve-MSCs; OPN-MSCs: osteopontin-preconditioned MSCs; F: female; M: male; N.A.: not applicable.

Experimental Group	Number of Animals Gene Expression			Number of Animals Histological Outcome		
	Total	Females	Males	Total	Females	Males
SHAM	7	3	4	13	6	7
HI VEH	9	4	5	23	10	13
HI N-MSC	N.A.			24	11	13
HI OPN-MSC	N.A.			22	10	12

## Data Availability

Data will be made available by the corresponding author upon request.

## Supplementary Materials

The following supporting information can be downloaded at https://www.mdpi.com/article/10.3390/cells14221824/s1, Table S1: Gene expression profile in the ipsilateral hemisphere at 3 days following neonatal hypoxic-ischemic brain injury.

## Abbreviations

The following abbreviations are used in this paper:

Bdnf	brain-derived neurotrophic factor
Cox-2	cycooxigenase-2
ERK	extracellular-signal-regulated kinase
FCS	fetal calf serum
Fgf-2	fibroblast growth factor 2
HI	hypoxia-ischemia/hypoxic-ischemic
HIE	hypoxic-ischemic encephalopathy
Il-6	interleukin-6
MSCs	mesenchymal stem cells
NGF	nerve growth factor
N-MSCs	naïve (non-preconditioned) MSCs
NSCs	neural stem cells
Opn	osteopontin
OPN-MSCs	osteopontin-preconditioned MSCs
SEM	standard error of the mean
Spp1	secreted phosphoprotein 1
Tgf-β	transforming growth factor β
Tnf-α	tumor necrosis factor α
Vegf	vascular endothelial growth factor
