# Supplementary material for: Osteopontin Preconditioning Improves the Regenerative Effects of Mesenchymal Stem Cells In Vitro but Not Their Therapeutic Efficacy Following Hypoxia-Ischemia in Mice
Source: Cells. 2025 Nov 20;14(22):1824. doi: 10.3390/cells14221824 (PMC12651268; doi:10.3390/cells14221824)
Supplement: Supplementary file 1 [file cells-14-01824-s001.zip › cells-3880829-supplementary.pdf]

Supplementary Materials

Table S1. Gene expression profile in the ipsilateral hemisphere at 3 days following neonatal hypoxic-ischemic brain injury. Gene expression profiles were examined using a pathway focused PCR array. Table shows genes which were differentially expressed after neonatal HI when compared to sham operated animals, and within the HI-injured brain in the ipsilateral hemisphere compared to the contralateral hemisphere . Numbers in red indicate more than 2 fold upregulated genes.

RefSeq	Symbol	Description	Fold regulation	
			Ipsilateral: HI vs SHAM	HI: ipsi- vs contra-lateral
NM_007445	*Amh*	Anti-Mullerian hormone	1.06	-1.64
NM_009711	*Artn*	Artemin	1.34	1.23
NM_007540	*Bdnf*	Brain derived neurotrophic factor	-1.27	-1.51
NM_009755	*Bmp1*	Bone morphogenetic protein 1	1.50	1.26
NM_009756	*Bmp10*	Bone morphogenetic protein 10	1.06	-1.64
NM_007553	*Bmp2*	Bone morphogenetic protein 2	1.18	-1.19
NM_173404	*Bmp3*	Bone morphogenetic protein 3	1.20	1.13
NM_007554	*Bmp4*	Bone morphogenetic protein 4	1.13	1.36
NM_007555	*Bmp5*	Bone morphogenetic protein 5	-1.02	-1.68
NM_007556	*Bmp6*	Bone morphogenetic protein 6	1.46	1.34
NM_007557	*Bmp7*	Bone morphogenetic protein 7	1.17	1.09
NM_007558	*Bmp8a*	Bone morphogenetic protein 8a	1.06	-1.64
NM_007559	*Bmp8b*	Bone morphogenetic protein 8b	1.16	-1.49
NM_007778	*Csf1*	Colony stimulating factor 1 (macrophage)	**3.21**	**3.43**
NM_009969	*Csf2*	Colony stimulating factor 2 (granulocyte-macrophage)	**5.15**	**4.62**
NM_009971	*Csf3*	Colony stimulating factor 3 (granulocyte)	1.06	-1.64
NM_008176	*Cxcl1*	Chemokine (C-X-C motif) ligand 1	1.06	-1.64
NM_021704	*Cxcl12*	Chemokine (C-X-C motif) ligand 12	-1.05	-1.17
NM_010113	*Egf*	Epidermal growth factor	1.61	1.41
NM_007950	*Ereg*	Epiregulin	1.06	-1.64
NM_010197	*Fgf1*	Fibroblast growth factor 1	1.29	1.12
NM_008002	*Fgf10*	Fibroblast growth factor 10	1.18	-1.01
NM_010198	*Fgf11*	Fibroblast growth factor 11	-1.23	-1.28
NM_010200	*Fgf13*	Fibroblast growth factor 13	-1.27	-1.57
NM_010201	*Fgf14*	Fibroblast growth factor 14	-1.11	-1.30
NM_008003	*Fgf15*	Fibroblast growth factor 15	**2.41**	1.73
NM_008004	*Fgf17*	Fibroblast growth factor 17	1.42	1.11

NM_008005	*Fgf18*	Fibroblast growth factor 18	1.17	-1.19
NM_008006	*Fgf2*	Fibroblast growth factor 2	1.58	1.90
NM_023304	*Fgf22*	Fibroblast growth factor 22	-1.16	1.53
NM_008007	*Fgf3*	Fibroblast growth factor 3	-1.36	-1.68
NM_010202	*Fgf4*	Fibroblast growth factor 4	1.06	-1.64
NM_010203	*Fgf5*	Fibroblast growth factor 5	-1.42	-1.96
NM_010204	*Fgf6*	Fibroblast growth factor 6	1.06	-1.64
NM_008008	*Fgf7*	Fibroblast growth factor 7	1.50	-1.16
NM_010205	*Fgf8*	Fibroblast growth factor 8	1.06	-1.64
NM_013518	*Fgf9*	Fibroblast growth factor 9	-1.26	-1.59
NM_010216	*Figf*	C-fos induced growth factor	1.11	1.06
NM_145741	*Gdf10*	Growth differentiation factor 10	1.16	-1.03
NM_010272	*Gdf11*	Growth differentiation factor 11	1.08	1.02
NM_008109	*Gdf5*	Growth differentiation factor 5	1.24	1.34
NM_010275	*Gdnf*	Glial cell line derived neurotrophic factor	-1.02	1.09
NM_010427	*Hgf*	Hepatocyte growth factor	1.92	1.51
NM_010512	*Igf1*	Insulin-like growth factor 1	**3.60**	**3.62**
NM_010514	*Igf2*	Insulin-like growth factor 2	1.44	1.91
NM_008350	*Il11*	Interleukin 11	1.06	-1.64
NM_008351	*Il12a*	Interleukin 12A	-1.27	-1.68
NM_008360	*Il18*	Interleukin 18	1.13	-1.12
NM_010554	*Il1a*	Interleukin 1 alpha	1.97	1.91
NM_008361	*Il1b*	Interleukin 1 beta	1.13	-1.03
NM_008366	*Il2*	Interleukin 2	1.06	-1.64
NM_010556	*Il3*	Interleukin 3	1.06	-1.64
NM_021283	*Il4*	Interleukin 4	1.06	1.23
NM_031168	*Il6*	Interleukin 6	1.06	-1.64
NM_008371	*Il7*	Interleukin 7	1.30	-1.44
NM_010564	*Inha*	Inhibin alpha	-1.08	1.05
NM_008380	*Inhba*	Inhibin beta-A	-1.54	-1.64
NM_008381	*Inhbb*	Inhibin beta-B	1.19	1.42
NM_013598	*Kitl*	Kit ligand	-1.17	-1.29
NM_010094	*Lefty1*	Left right determination factor 1	-1.13	-1.51
NM_177099	*Lefty2*	Left-right determination factor 2	1.31	1.32
NM_008493	*Lep*	Leptin	1.06	-1.64

NM_008501	*Lif*	Leukemia inhibitory factor	1.06	-1.64
NM_010784	*Mdk*	Midkine	1.62	1.44
NM_010834	*Mstn*	Myostatin	1.27	1.31
NM_013609	*Ngf*	Nerve growth factor	-1.30	-1.57
NM_013611	*Nodal*	Nodal	1.06	-1.64
NM_008742	*Ntf3*	Neurotrophin 3	-1.20	-1.40
NM_198190	*Ntf5*	Neurotrophin 5	1.06	-1.64
NM_008808	*Pdgfa*	Platelet derived growth factor, alpha	1.12	-1.16
NM_008827	*Pgf*	Placental growth factor	1.31	1.40
NM_019400	*Rabep1*	Rabaptin, RAB GTPase binding effector protein 1	-1.08	-1.51
NM_011313	*S100a6*	S100 calcium binding protein A6 (calcyclin)	**5.03**	**4.33**
NM_009263	*Spp1*	Secreted phosphoprotein 1	**194.10**	**65.06**
NM_011562	*Tdgf1*	Teratocarcinoma-derived growth factor 1	**2.84**	1.60
NM_009362	*Tff1*	Trefoil factor 1	1.06	-1.64
NM_031199	*Tgfa*	Transforming growth factor alpha	1.05	-1.05
NM_011577	*Tgfb1*	Transforming growth factor, beta 1	**2.42**	1.80
NM_009367	*Tgfb2*	Transforming growth factor, beta 2	1.07	1.09
NM_009368	*Tgfb3*	Transforming growth factor, beta 3	1.57	1.66
NM_009505	*Vegfa*	Vascular endothelial growth factor A	-1.22	-1.49
NM_011697	*Vegfb*	Vascular endothelial growth factor B	-1.01	-1.08
NM_009506	*Vegfc*	Vascular endothelial growth factor C	-1.49	-1.22
NM_053009	*Zfp91*	Zinc finger protein 91	1.17	1.12
NM_007393	*Actb*	Actin, beta	1.54	1.45
NM_009735	*B2m*	Beta-2 microglobulin	**2.86**	**2.62**
NM_008084	*Gapdh*	Glyceraldehyde-3-phosphate dehydrogenase	-1.01	1.05
NM_010368	*Gusb*	Glucuronidase, beta	**4.31**	**3.71**
NM_008302	*Hsp90ab1*	Heat shock protein 90 alpha (cytosolic), class B member 1	1.01	-1.05
